# A serosurvey of selected cystogenic coccidia in Spanish equids: first detection of anti-*Besnoitia* spp. specific antibodies in Europe

**DOI:** 10.1186/s12917-017-1046-z

**Published:** 2017-05-10

**Authors:** Daniel Gutiérrez-Expósito, Ignacio García-Bocanegra, Daniel K. Howe, Antonio Arenas-Montes, Michelle R. Yeargan, SallyAnne L. Ness, Luis M. Ortega-Mora, G. Álvarez-García

**Affiliations:** 1 SALUVET, Animal Health Department, Faculty of Veterinary Sciences, Complutense University of Madrid, Ciudad Universitaria s/n, 28040 Madrid, Spain; 2 Animal Health Department, Faculty of Veterinary Sciences, University of Cordoba-Agrifood Excellence International Campus (ceiA3), Cordoba, Spain; 3 Department of Veterinary Science, M.H. Gluck Equine Research Center, University of Kentucky, Lexington, KY 40546-0099 USA; 4 Department of Clinical Sciences, College of Veterinary Medicine, Box 52, Cornell University, Ithaca, NY 14853 USA

**Keywords:** *Besnoitia* spp., *Sarcocystis* spp., *Neospora* spp., Spain, Serosurvey, Horse, Donkey, Mule, Risk factors

## Abstract

**Background:**

Equine besnoitiosis, caused by *Besnoitia bennetti,* and equine protozoal myeloencephalitis (EPM), caused by *Sarcocystis neurona* and *Neospora hughesi* are relevant equine diseases in the Americas that have been scarcely studied in Europe. Thus, a serosurvey of these cystogenic coccidia was carried out in Southern Spain. A cross-sectional study was performed and serum samples from horses (*n* = 553), donkeys (*n* = 85) and mules (*n* = 83) were included. An *in-house* enzyme-linked immunosorbent assay (ELISA) was employed to identify a *Besnoitia* spp. infection and positive results were confirmed by an a posteriori western blot. For *Neospora* spp. and *Sarcocystis* spp., infections were detected using *in-house* ELISAs based on the parasite surface antigens *N. hughesi* rNhSAG1 and *S. neurona* rSnSAG2/3/4. Risk factors associated with these protozoan infections were also investigated.

**Results:**

Antibodies against *Besnoitia* spp., *Neospora* spp. and *Sarcocystis* spp. infections were detected in 51 (7.1%), 46 (6.4%) and 20 (2.8%) of 721 equids, respectively. The principal risk factors associated with a higher seroprevalence of *Besnoitia* spp. were the host species (mule or donkey), the absence of shelter and the absence of a rodent control programme. The presence of rodents was the only risk factor for *Neospora* spp. infection.

**Conclusions:**

This study was the first extensive serosurvey of *Besnoitia* spp. infection in European equids accomplished by two complementary tests and gives evidence of the presence of specific antibodies in these populations. However, the origin of the infection is still unclear. Further parasite detection and molecular genotyping are needed to identify the causative *Besnoitia* and *Neospora* species. Finally, cross-reactions with antibodies directed against other species of *Sarcocystis* might explain the positive reactions against the *S. neurona* antigens.

**Electronic supplementary material:**

The online version of this article (doi:10.1186/s12917-017-1046-z) contains supplementary material, which is available to authorized users.

## Background

Cystogenic coccidia such as *Sarcocystis* spp., *Neospora* spp., and *Besnoitia* spp. have been reported to affect equids. *Sarcocystis neurona* and *Neospora hughesi* are the causative agents of equine protozoal myeloencephalitis (EPM), a serious neurological disease of horses in the Americas [1]. Moreover, besnoitiosis in donkeys caused by *Besnoitia bennetti* in donkeys is an emerging disease in the United States [2].

Several serosurveys of *Sarcocystis* spp. and/or *Neospora* spp. infections have been carried out in horses and donkeys in Europe (France, Italy, Czech Republic, Sweden and Spain) [3, 4]. However, *Besnoitia* spp. infection has not been studied in depth in European equids apart from only two reports of equine besnoitiosis. The first case of besnoitiosis in a horse was reported in Northern France [5]. Recently, the disease was suspected in seven donkeys from Southern Spain since tissue cysts were detected by histopathology [6]. Apart from *B. bennetti*, two additional *Besnoitia* species (*B. besnoiti* and *B. tarandi*) also affect ungulates (i.e., bovines and cervidae) causing similar clinical signs and have been reported in Europe [7]. Besnoitiosis caused by *B. tarandi* has been documented in reindeer in the Artic regions [8], whereas besnoitiosis caused by *B. besnoiti* is a re-emergent cattle disease in western and Central Europe and has also been recently reported in roe deer and red deer in Spain [9–11].

Diagnostic tools that provide an accurate serological diagnosis of cystogenic coccidia infections must overcome cross-reactions. Particularly in equids, *N. hughesi* cross-reacts with *N. caninum* [12]. In addition, cross-reactions between anti-*B. bennetti* antibodies and *B. besnoiti* antigens have also been observed [13]. Thus, highly sensitive and specific tests are mandatory in order to confirm an infection. Enzyme-linked immunosorbent assays (ELISAs)-based on recombinant proteins provide an accurate diagnosis of *S. neurona* and *N. hughesi* infection [14, 15]. A *B. besnoiti* tachyzoite extract-based ELISA is a routinely employed screening technique for *Besnoitia* spp. infection and a western blot is used as a confirmatory test in various ungulate species [2, 16]. In addition, a novel ELISA based on the enrichment of specific antigens has been demonstrated to be highly specific for the diagnosis of bovine besnoitiosis [17].

The goal of the present work was to determine the presence of specific antibodies against *Besnoitia* spp., *Neospora* spp. and *Sarcocystis* spp. in horses, donkeys and mules from southern Spain (Andalusia). This was the first serosurvey of *Besnoitia* spp. infection in European equids. Moreover, the first results of anti-*Neospora* spp. antibodies detection in Spanish equids are presented.

## Methods

### Sampled areas and experimental design

A cross-sectional study was carried out between January and March of 2010 in equine herds from Andalusia (southern Spain; 36° N - 38° 60′ N, 1° 75′ W - 7° 25′ W), which is the Spanish region with the largest number of equines (see the discussion section).

**Fig. 1:**
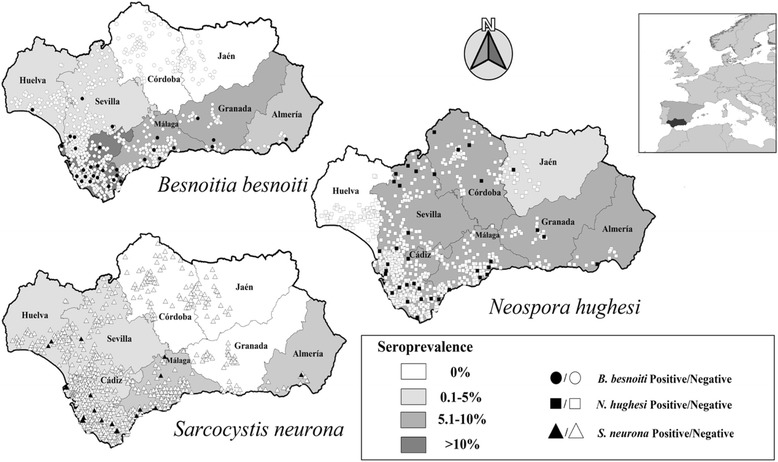
Geographical distribution of equids sampled in Andalusia and seropositive results to *B. besnoiti*, *N. hughesi* and *S. neurona* infections. Positive results are marked in *black* and negative results are marked in *white*. Prevalence of infection by province is represented in *grey*

A total of 721 samples from horses (*n* = 553), donkeys (*n* = 85) and mules (*n* = 83) were included. Specifically, samples from 616 horses were collected in a survey stratified by census tract. We used a convenience sampling technique to select the remaining samples. Horse samples were distributed across all provinces of Andalusia, whereas the donkey and mule samples were restricted to Cádiz Province (Fig. 1). ELISAs based on *S. neurona* and *N. hughesi* recombinant proteins were employed for the detection of anti-*Sarcocystis* spp. and anti-*Neospora* spp. antibodies. To detect anti-*Besnoitia* spp. antibodies, all serum samples were initially screened by a soluble extract-based ELISA, and positive results were confirmed by western blot. Animals seropositive according to the western blot were included in the data analysis.

### Samples and data collection

Blood samples were collected by puncturing of the jugular vein using a sterile collection system (Vacutainer®, Becton, Dickinson and Company, USA). Next, the blood samples were centrifuged at 400 *g* for 15 min at 4 °C and the sera were separated and stored at −20 °C until further analysis.

Epidemiological data were collected by an on-farm interview with the owners (Additional file 1), who were informed of the goals of the study. The questionnaires were especially designed to collect information using “close-ended” questions to avoid ambiguous or lengthy answers. In total, 18 explanatory variables were included in the analysis: species (horse, mule and donkey), age classes (young: < 5 years, adult: 5–16 years and geriatric: > 16 years), gender (male and female), colour (dark and light), breed (Spanish, Arabian, Spanish-Arabian, other purebred and crossbred), province, activity (farming, leisure and work), type of housing (outside and individual or collective shelter), direct contact with other horses, mules or donkeys, presence of other animal species (domestic and wild birds, domestic and wild ruminants), presence of rodents, insecticide treatment, cleaning and disinfection methods and protocols, pest control programmes (insects and rodents) and water sources. The sero-status of any animals that shared the same habitat as the sampled equids was unknown.

### Antigen production for serological tests

Culture-derived tachyzoites of *B. besnoiti* isolate -Bb-Spain 1 [18] were propagated and purified [19] to prepare antigens for the ELISA and western blot tests. Foetal bovine serum was previously checked for the absence of anti-*Besnoitia*, anti-*N. caninum* and anti-*T. gondii* antibodies by an immunofluoresce antibody test (IFAT) [18]. Soluble *B. besnoiti* tachyzoite antigens used in the ELISA were prepared and quantified as previously described [20] and maintained at −80 °C until use. *Besnoitia besnoiti* tachyzoites were pelleted and frozen at −80 °C for western blot.

*Sarcocystis neurona* and *N. hughesi* recombinant proteins were purified as described previously [14, 15] and maintained at 4 °C until use.

### Serology

#### ELISAs

**Fig. 2:**
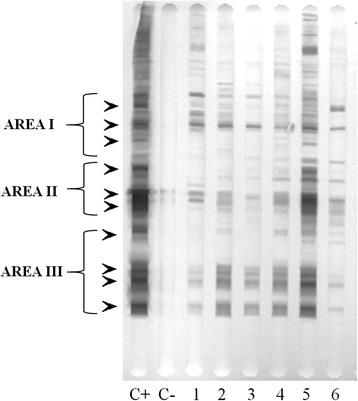
Recognition of *Besnoitia* spp. tachyzoite antigens by western blot. Lines 1–6: samples from *Besnoitia* spp*.* ELISA seropositive equids. *Arrows* indicate recognition of antigenic bands in each of the three principal antigenic areas

*Besnoitia besnoiti* tachyzoite soluble extract was used as antigens because strong cross-reactions were present between *B. besnoiti* antigens and anti-*B. bennetti* specific antibodies as indicated by IFAT and western blot [13]. Serum samples were analysed in duplicate, as previously described [21], with a few modifications: (i) a blocking solution of phosphate buffered saline (PBS) containing 0.05% Tween 20 and 3% bovine serum albumin (Roche®) was used, and (ii) a rabbit peroxidase-labelled anti-horse IgG (H + L) antibody conjugate (INGENASA®) diluted at 1:5000 was used. The cut-off value was selected on the basis of three standard deviations of optical density (OD) values obtained with a panel of seronegative horse samples (*n* = 20). Positive and negative control sera tested by western blot consisted of chronically infected donkeys [13] and non-infected horses from USA and Spain, respectively. A cut-off at OD values higher than 0.40 was established and ELISA-positive results were confirmed by a western blot (Fig. 2).

Anti-*Neospora* spp. and anti-*Sarcocystis* spp. antibodies were detected using the recombinant *N. hughesi* surface antigen rNhSAG1 and *S. neurona* trivalent protein rSnSAG2/4/3 by means of previously described ELISAs [14, 15]. Percent positivity (PP) values of 20 and 15 were used as cut-offs for *N. hughesi* and *S. neurona* antigen-based ELISAs, respectively. The horse positive control serum employed in the *Neospora* spp.-based ELISA came from a mare that was experimentally infected during pregnancy with *N. hughesi*. The horse positive control serum for the *Sarcocystis* spp.-based ELISA was collected from two clinically affected horses with confirmed EPM by histopathology. The negative control serum for both ELISAs consisted of a serum collected from a weanling prior to infection with *S. neurona* [22]. This serum was negative by *S. neurona*-, *T. gondii*-, *N. caninum*-, and *N. hughesi*- based western blots.

### SDS-PAGE and western blot

A total of 4 × 10^7^ tachyzoites under non-reducing conditions were employed for the electrophoresis of *B*. *besnoiti* [23]. Tachyzoite antigens were transferred to a nitrocellulose membrane and, incubated with sera from either horses, donkeys or mules at a 1:20 dilution, followed by a peroxidase-conjugated anti-horse IgG (H + L) antibody diluted at 1:500 (INGENASA®, Madrid, Spain). Control sera were the same as used for the ELISA. The presence of at least three bands in at least two of the three principal antigenic areas (area I: 72.5, 58.9 and 51.4 kDa; area II: 38.7, 31.8 and 28.5 kDa; area III: 23.6, 19.1, 17.4, 14.5 kDa) was considered as a positive result for a *Besnoitia* spp. infection (Fig. 2) [23].

### Statistical analysis

The prevalence of antibodies against *Besnoitia* spp.*, Neospora* spp. and *Sarcocystis* spp. infections was estimated with the exact binomial confidence intervals of 95% [24].

To detect non-linear relationships and to homogenize the scales of the explanatory variables, all quantitative variables were transformed to qualitative variables using three categories of the 33rd and 66th percentiles as cut-off values. Tests of association were performed in three steps. First, a general linear univariate analysis was performed. The herd was the experimental unit, and the individual seroprevalence against *B. besnoiti* and *N. hughesi* was the dependent variable. Second, factors showing a *P-value* < 0.15 were further scrutinized for associations using Spearman’s rank correlation coefficient (*r*) to avoid colinearity problems. When colinearity (*P* < 0.05 and *r* > 0.4) was present, only the variable more clearly linked to *Besnoitia* spp. and *Neospora* spp. seropositivity was retained. The third step involved a generalized estimating equations model (GEE) [25]. The number of seropositive animals was assumed to follow a binomial distribution and the herd was included as a random effect. A Poisson error distribution and a logit link function were considered.

An initial model was obtained using all of the potential explanatory variables and variables with a non-significant *P*-value were sequentially deleted. The quasi-likelihood under an independence model criterion was used to determine the best model in terms of its potential for explaining the results. Biologically plausible confounding factors were assessed using a Mantel-Haenszel analysis and confounding was considered to be potentially significant if the odds ratios (ORs) were shifted appreciably. Variables that altered the coefficients of the independent variables of interest by 30% or more when removed from the model were classified as confounding factors. The model was re-run until all of the remaining variables were statistically significant (i.e., the likelihood-ratio via Wald’s test had *P* < 0.05), and a potential causal relationship with the response variable existed. SPSS 22.0 software (SPSS Inc., Chicago, IL, USA) was used for statistical analysis.

The association between *Besnoitia* spp. ELISA false-positive results and the presence of anti-*Neospora* spp. and anti-*Sarcocystis* spp. antibodies was estimated by using the chi-square test. A Mann–Whitney *U* test was used to compare differences in the anti-*Besnoitia* spp. antibody levels estimated by ELISA between the false-positive and true-positive results. These statistical analyses were performed with the InStat 3.05 software (GraphPad). Additionally, the test agreement expressed as the kappa-values (*k*) between the ELISA and the western blot tests was calculated using WinEpiscope 2.0 [26].

## Results

### Seroprevalence

Antibodies against *Besnoitia* spp., *Neospora* spp. and *Sarcocystis* spp. were detected in 51 (7.1%; CI_95%_: 5.2–8.9), 46 (6.4%; CI_95%_: 4.6–8.2) and 20 (2.8%; CI_95%_: 1.6–4.0) of 721 equids tested, respectively. Seropositivity against both *Besnoitia* spp. and *Neospora* spp. were confirmed in 0.8% (6/721), against both *Besnoitia* spp. and *Sarcocystis* spp. in 0.6% (4/721) and against both *Neospora* spp. and *Sarcocystis* spp. in 0.4% (3/721) equids (Table 1). A good agreement between the ELISA and the western blot tests was obtained (*k* = 0.6).

**Table 1:** Variables identified as significant (*P* < 0.15 in the univariate analysis) and included in the multivariate analysis

			*Besnoitia besnoiti*		*Neospora hughesi*	
Variables	Categories	No. analyzed ^a^	Positive (%)	*P*-value	Positive (%)	*P*-value
Species	Horse	553	16 (2.9)	<0.001		
	Donkey	85	13 (15.3)			
	Mule	83	22 (26.5)			
Breed	Pure	370	18 (4.9)	0.005	30 (8.1)	0.021
	Crossbred	298	31 (10.4)		12 (4.0)	
Sex	Female	306	26 (8.5)	0.129		
	Male	415	25 (6.0)			
Province	Almeria	14	1 (7.1)	<0.001		
	Cadiz	320	40 (12.5)			
	Córdoba	60	0 (0.0)			
	Granada	38	3 (7.9)			
	Huelva	60	1 (1.7)			
	Jaen	30	0 (0.0)			
	Malaga	98	5 (5.1)			
	Seville	101	1 (1.0)			
Presence of shelters	Yes	407	15 (3.7)	<0.001	23 (5.7)	0.122
	No	280	35 (12.5)		23 (8.2)	
Presence of wild birds	Yes	403	40 (9.9)	0.001		
	No	275	9 (3.3)			
Presence of cattle	Yes	145	19 (13.1)	0.020	17 (11.7)	0.017
	No	417	29 (7.0)		24 (5.8)	
Presence of wild ruminants	Yes	41			8 (19.5)	0.006
	No	521			33 (6.3)	
Presence of rodents	Yes	388	35 (9.0)	0.001	34 (8.8)	0.002
	No	261	8 (3.1)		15 (5.7)	
Rodent control program	Yes	434	24 (5.5)	0.009		
	No	226	25 (11.1)			
Cleaning protocol	Yes	581	38 (6.5)	0.018	32 (5.5)	0.068
	No	86	12 (14.0)		9 (10.5)	
Disinfection protocol	Yes	536	32 (6.0)	0.006		
	No	127	17 (13.4)			

^a^Missing values were omitted

### Cross-reactions between anti-Besnoitia spp. antibodies and other cystogenic coccidia

Antibodies against *Neospora* spp. and/or *Sarcocystis* spp. were detected in 8.7% (63/721) of the sampled animals (Table 2). Interestingly, true *Besnoitia*-seropositive animals that were also positive against *Neospora* spp. and/or *Sarcocystis* spp. had OD values higher than 0.8, whereas OD values of false *Besnoitia*-seropositive animals varied from 0.40 to 0.56 (Table 2) (*P* < 0.001, Mann–Whitney *U* test). However, the existence of *Besnoitia* spp. false-positive results was not significantly associated with seropositivity against *Neospora* spp. or *Sarcocystis* spp. (*P* = 0.55).

**Table 2:** Detection of anti-*Besnoitia* spp., anti-*Sarcocystis* spp. and anti-*Neospora* spp. antibodies by ELISA

	*n*	Seropositive to *Sarcocystis* spp. (%)	Seropositive to *Neospora* spp. (%)	Seropositive to *Sarcocystis* spp. and *Neospora* spp. (%)	Seronegative to *Sarcocystis* spp. and *Neospora* spp. (%)
*Besnoitia* spp. seropositive^a^	51	4 (7.84%)	6 (11.76%)	0 (0.0%)	41 (80.39%)
*Besnoitia* spp. seronegative	641	11 (1.71%)	36 (5.61%)	1 (0.15%)	593 (92.51%)
*Besnoitia* spp. seronegative with a false-positive ELISA result	29	2 (6.89%)	1 (3.44%)	2 (6.89%)	24 (82.75%)
Total	721	17 (2.35%)	43 (5.96%)	3 (0.41%)	658 (91.26%)

^a^True *Besnoitia*-seropositive animals confirmed by western blot

### Risk factors

Due to the low number of *Sarcocystis* spp. seropositive animals, risk factors were only analysed using *Besnoitia* spp. and *Neospora* spp. seropositive animals as the dependent variables.

Eleven explanatory variables were selected from the univariate analysis for *Besnoitia* spp. infection (*P* < 0.15) (Table 2). The seroprevalence was significantly higher in mules (26.5%) and donkeys (13.3%) compared to that in horses (2.9%) (Table 2). The seroprevalence values differed among provinces for horses (Fig. 1) and the seroprevalence was lower in the presence of shelters, whereas it was higher in the presence of rodents (Table 2).

The principal risk factors associated with a *Besnoitia* spp. infection were species (mule and donkey) (OR = 12.06 and OR = 2.06, respectively), the absence of shelters (OR = 2.45) and the absence of rodent a control programme (OR = 5.34) (Table 3). The presence of rodents (OR = 4.83) was the only risk factor for a *Neospora* spp. infection (Table 3).

**Table 3:** Generalized estimating equations model of potential risk factors associated with *Besnoitia* spp. and *Neospora* spp. seropositivity

Variable	Category	*β*	Sig.	OR	95% CI	
*Besnoitia* spp.						
Species	Horse	*	*	*	*	*
	Donkey	0.723	0.116	2.060	0.836	5.075
	Mule	2.490	<0.001	12.061	3.907	37.235
Presence of shelters	Yes	*	*	*	*	*
	No	0.898	0.038	2.455	1.053	5.725
Rodent control program	Yes	*	*	*	*	*
	No	1.676	0.001	5.342	1.952	14.623
*Neospora* spp.						
Presence of rodents	Yes	1.575	0.003	4.831	1.680	13.890
	No	*	*	*	*	*

* Reference category; OR, Odds ratio; 95% CI, 95% Confidence interval

## Discussion

In the present study, we investigated three cystogenic coccidial infections that have been little studied in European equids. In fact, clinical cases of EPM have not been diagnosed in the Old World. Regarding other epidemiological gaps, the definitive hosts of *N. hughesi* and *Besnoitia* spp. that affect ungulates are still unknown [7, 27]. In contrast, the definitive host of *S. neurona* (*Didelphis* spp.) appears to be restricted to the New World, but other species of *Sarcocystis* are known to infect equids in the Old World [4].

This serosurvey was focused on Andalusia, which has the highest number of equid herds in Spain (44.3%) [28]. In addition, *Besnoitia* spp. infections have been reported in cattle and suspected in donkeys from Andalusia [6, 29]. In contrast, no data exist for *Neospora* spp. and *Sarcocystis* spp. infections in equids from this region. Thus, the impact of EPM is unknown.

The most relevant finding of this work was the first detection of specific antibodies against *Besnoitia* spp. in European equids. The diagnostic approach (i.e., an initial screening by ELISA followed by a confirmatory western blot) was previously used in serosurveys conducted in the absence of a panel of reference sera and a gold standard test [11, 16] using sera from clinically *Besnoitia* spp.-infected donkeys as a positive control [13, 23]. Additionally, serological cross-reactions with antibodies against *Neospora* spp. and *Sarcocystis* spp. were ruled out, probably due to the low antibody levels detected against *Neospora* spp. and *Sarcocystis* spp. in this study and the use of highly specific recombinant proteins. Since the *Besnoitia* ELISA can yield ambiguous results, a western blot or a more specific ELISA are mandatory in order to confirm the infection [17].

Outside of Europe, a few cases of *Besnoitia* spp. infections in various equid species (horse, donkey, mule and zebra) were attributed to *B. bennetti* infection in the mid-twentieth century in different sub-Saharan countries such as South Africa and Sudan [30–32]. It has been suggested that besnoitiosis may be an emerging disease in donkeys in the United States [2]. Furthermore, equine besnoitiosis in Europe was first reported in 1922 [5] in a horse in Northern France. The disease has not been diagnosed since, but in a recent outbreak of donkeys from Southern Spain besnoitiosis was suspected [6]. Seroprevalence rates of *Besnoitia* spp. infection in other ungulate species of Spain of 1% (2/2608) and 0% (0/2285) have been reported in wild and small domestic ruminants, respectively, in areas where bovine besnoitiosis is highly endemic [11, 33]. However, whether equids may be reservoirs or intermediate hosts of *B. besnoiti* should be further elucidated through molecular genotyping [34].

Interestingly, the highest seroprevalence of *Besnoitia* spp. infection was observed in donkeys and mules. However, a higher susceptibility to *Besnoitia* spp. infection in donkeys and mules compared to that in horses has not been demonstrated. Most of the clinical cases of equine besnoitiosis have been reported in donkeys [2, 35, 36]. Although less frequently, the disease has also been diagnosed in horses [30, 32, 37]. The differences in the management and biosecurity measures between donkeys/mules and horse herds could be a feasible explanation for this finding. Similar to bovine besnoitiosis, parasite transmission might occur through direct contact during natural mating or from bites of blood-sucking arthropods, and animal movement might favour the spread of the disease [29]. Indeed, it was suggested that the use of repellents indoors may help to reduce mechanical transmission by blood-sucking insects [29]. In this study, data on treatment with ectoparasiticides were only recorded in 229 animals and 190 were treated (156 of the treated animals were horses; data not shown). In addition, the absence of shelters would also lead to a higher exposure to blood-sucking arthropods. Finally, whether rodents may act as intermediate hosts of *B. bennetti* as they do for other *Besnoitia* species (*B. akodoni, B. jellisoni, B. neotomofelis* and *B. wallacei*) has not been clarified yet [7, 9]. The *Besnoitia* species that parasitize equids must be elucidated. Because *B. besnoiti* and *B. bennetti* infections are serologically indistinguishable, further molecular analyses and parasite isolations are necessary in order to determine which *Besnoitia* species might be present in Spanish equids.

Antibodies against the aetiological agents of EPM (*N. hughesi* and *S. neurona*) were also researched in this study. However, the simple detection of a specific antibody does not confirm the diagnosis of the disease since EPM occurs in a small proportion of infected horses [1]. This initial serosurvey of *Neospora* spp. infection carried out in Spanish equids showed a low seroprevalence (6.4%) and that the presence of rodents was a risk factor for a *Neospora* spp. infection. These results agreed with other studies carried out in different European countries such as France, Sweden and Italy that revealed a seroprevalence of approximately 10% [3, 38, 39]. Little it is known about the role of rodents in the epidemiology of equine neosporosis. Rodents could be considered as a putative intermediate host of equine neosporosis, similarly to bovine neosporosis. In fact, *N. caninum* DNA could be detected in mice and rats in cattle herds with a previous history of neosporosis [40–42]. Equids likely become infected with *Neospora* spp. via the ingestion of oocysts shed by the definitive host, or possibly through vertical transmission [43, 44].

The *Neospora* species present in these equids remain to be elucidated, since cross-reactions between both existing *Neospora* species are widely known [12]. Indeed, the rNhSAG1-based ELISA clearly detects antibodies to the *N. caninum* orthologue [14]. In this study, *Neospora* spp. seropositive sera were demonstrated to be seropositive by *N. caninum*-based western blot, supporting this assumption (see Additional file 2).

Finally, anti-*Sarcocystis* spp. antibodies were detected in 2.8% equids and this level is similar to previous reports in France and northern Spain [45, 46]. Spanish horses are unlikely to be exposed to *S. neurona* due to the absence of the definitive host in Europe but cross-reactions with other species of *Sarcocystis* might explain positive reactions. In fact, the trivalent rSnSAG surface antigen chimaera-based ELISA was not validated with sera from European equids. Additionally, other latent *Sarcocystis* spp. cysts are present in equids and are often benign.

## Conclusions

This is the first serosurvey of *Besnoitia* spp. and *Neospora* spp. infections carried out in Spain. This study found specific antibodies against *Besnoitia* spp. infection in European equids. Anti-*Neospora* spp. and *Sarcocystis* spp. antibodies were also found in a few animals. However, neither parasitic disease had been not previously diagnosed in Europe. Further parasite detection and molecular genotyping are needed to clarify the presence and identity of these three parasite species [11].

## Additional files

Additional file 1: Questionnaire survey developed for use in this study. (DOCX 129 kb)

Additional file 2: Detection of specific anti-*Neospora* spp. antibodies by a western blot test validated for bovine neosporosis [47]. (DOC 302 kb)

## Abbreviations

B: Besnoitia
ELISA: Enzyme-linked immunosorbent assay
EPM: Equine protozoal myeloencephalitis
GEE: Generalized estimating equation
IFAT: Immunofluorescence antibody test
Ig: Immunoglobulin
k: Kappa value
kDa: Kilodalton
N: *Neospora*
OD: Optical density
OR: Odd ratio
S: *Sarcocystis*
SPSS: Statistical package for the social sciences
