# Supplementary material for: A serosurvey of selected cystogenic coccidia in Spanish equids: first detection of anti-Besnoitia spp. specific antibodies in Europe
Source: BMC Vet Res. 2017 May 10;13:128. doi: 10.1186/s12917-017-1046-z (PMC5424396; doi:10.1186/s12917-017-1046-z)
Supplement: Supplementary file 1 — Questionnaire survey developed for use in this study. (DOCX 129 kb) [file 12917_2017_1046_MOESM1_ESM.docx]

**Date: ......./......../........ Questionnaire number: .......... Sample number:**

**Town: Province:**

**1. PERSONAL DATA**

Veterinarian: ___________________ Phone: ____________

Owner: _____________________________

**2. INDIVIDUAL DATA**

**Species**:  Horse  Mule  Donkey Age (years): _________________

**Breed**:  Spanish  Arabian Spanish-arabian  Other pure breed  Crossbreed

**Colour:**  Dark  Light

**Sex**:  Female  Male  Castrated male

**Activity**:  Farming  Work  Leisure  Others: ………

**Overall status**:  Good  Regular  Poor

**Recent diseases**:  No  Yes (explain): ………………………………….

**Vaccination programme:**  No  Yes (explain): ………………………………….

**Desparasitization programme**:  No  Yes (explain): ……………………………

**Insecticide treatment**:  No  Yes (explain): ……………………………

**3. FARM DATA**

**Type of housing:**

 Outside  Individual shelter  Collective shelter

**Water source:**  Well Water network

**Species in the farm**

Horses:……… Cats:……… Dogs:……… Others: ………

**Contact with equids outside the farm** No Yes

**Contact with wild animals:** No Yes (species):

**Presence of rodents:** No Yes

**Food**:  Feed  Hay  Straw  Alfalfa  Others: …………

**Cleaning protocol**  once a week  twice a week  three times a week

**Disinfection protocol**  once a week  twice a week  three times a week

**Rodent control**  No  Yes (explain):

**Fumigation control**  No  Yes (explain):

**Comments:**
