# Supplementary material for: A serosurvey of selected cystogenic coccidia in Spanish equids: first detection of anti-Besnoitia spp. specific antibodies in Europe
Source: BMC Vet Res. 2017 May 10;13:128. doi: 10.1186/s12917-017-1046-z (PMC5424396; doi:10.1186/s12917-017-1046-z)
Supplement: Supplementary file 2 — Detection of specific anti-Neospora spp. antibodies by a western blot test validated for bovine neosporosis [47]. (DOC 302 kb) [file 12917_2017_1046_MOESM2_ESM.doc]

**Additional file 2**

**Detection of specific anti-*Neospora* spp. antibodies by a western blot test validated for bovine neosporosis**

Culture-derived tachyzoites of *N. caninum* isolate Nc-1 [47] were propagated and purified [19] and subsequently pelleted and frozen at -80 ºC. Fetal bovine serum was previously checked for absence of anti-*Besnoitia*, anti-*N. caninum* and anti-*T. gondii* IgG by IFAT [21]. A total of 2x10^7^ *N.caninum*-tachyzoites under reducing conditions were employed for electrophoresis [20]. Tachyzoite antigen was transferred to membranes, then incubated with sera from either horses, donkeys, mules or cattle at 1:20 dilution, followed by a peroxidase-conjugated anti-horse IgG (H+L) antibody diluted at 1:1000 (INGENASA^®^) for equids species and a mouse monoclonal anti-bovine IgG1/IgG2 peroxidase-conjugated diluted at 1:1200 (Thermo Fisher Scientific, EE.UU) for cattle. Sera from a *N. caninum*-infected cow [20] and a *N. hughesi*-infected horse [14] were used as positive control in order to compare both pattern recognitions. The recognition of the 17-18, 34-35, 37 and 60-62 kDa immunodominant antigens (IDAs) was considered as a positive reaction [20].

In order to confirm *Neospora* spp. infection, ELISA-positive results were re-tested by a *N. caninum* tachyzoite-based western blot. Thirty-seven out of 46 ELISA-positive results were also positive against *N. caninum* tachyzoite-based western blot and minor differences were observed in the pattern of recognition of equids and cattle (see Figure below).

Figure: Recognition of *Neospora* spp. tachyzoite antigens by a *N. caninum* tachyzoite-based western blot. Lines 1-7: ELISA-positive samples. Lines 8-9: ELISA-negative samples. Line 10: Control positive serum of horse. Line 11: Control positive serum of cattle. Arrows indicate recognition of IDAs.
